# Leukocyte immunoglobulin-like receptor B4 regulates key signalling molecules involved in FcγRI-mediated clathrin-dependent endocytosis and phagocytosis

**DOI:** 10.1038/srep35085

**Published:** 2016-10-11

**Authors:** Mijeong Park, Mark J. Raftery, Paul S. Thomas, Carolyn L. Geczy, Katherine Bryant, Nicodemus Tedla

**Affiliations:** 1 Inflammation and Infection Research Centre, School of Medical Sciences, Faculty of Medicine, University of NSW, Sydney, NSW 2052, Australia; 2 Bioanalytical Mass Spectrometry Facility, Department of Medicine, University of NSW, Sydney, NSW 2052, Australia; 3 Department of Respiratory Medicine, Prince of Wales Hospital, Sydney, NSW 2031, Australia

## Abstract

FcγRI cross-linking on monocytes may trigger clathrin-mediated endocytosis, likely through interaction of multiple intracellular molecules that are controlled by phosphorylation and dephosphorylation events. However, the identity of phospho-proteins and their regulation are unknown. We proposed the leukocyte immunoglobulin-like receptor B4 (LILRB4) that inhibits FcγRI-mediated cytokine production via Tyr dephosphorylation of multiple kinases, may also regulate endocytosis/phagocytosis through similar mechanisms. FcγRI and/or LILRB4 were antibody-ligated on THP-1 cells, lysates immunoprecipitated using anti-pTyr antibody and peptides sequenced by mass spectrometry. Mascot Search identified 25 Tyr phosphorylated peptides with high confidence. Ingenuity Pathway Analysis revealed that the most significantly affected pathways were clathrin-mediated endocytosis and Fc-receptor dependent phagocytosis. Tyr phosphorylation of key candidate proteins in these pathways included common γ-chain of the Fc receptors, Syk, clathrin, E3 ubiquitin protein ligase Cbl, hepatocyte growth factor-regulated tyrosine kinase substrate, tripartite motif-containing 21 and heat shock protein 70. Importantly, co-ligation of LILRB4 with FcγRI caused significant dephosphorylation of these proteins and was associated with suppression of Fc receptor-dependent uptake of antibody-opsonised bacterial particles, indicating that LILRB4. These results suggest that Tyr phosphorylation may be critical in FcγRI-dependent endocytosis/phagocytosis that may be regulated by LILRB4 by triggering dephosphorylation of key signalling proteins.

Fc receptors (FcRs) are key molecules for recognition and elimination of foreign antigens through induction of multiple inflammatory mediators and antigen presentation^1^. FcγRI (CD64) expressed on mono-myeloid cells^2^,^3^ is a high affinity receptor for monomeric IgG. Cross-linking of FcγRI by immune complexes initiates cellular responses and internalization of the receptor/ligand(s)^1^,^4^. The phospho-signalling mechanisms in FcγRI-mediated activation leading to cytokine release and/or induction of the oxidative burst are well recognised^5^. In contrast, events following FcγRI receptor internalization after cross-linking of immune complexes are less well defined, although two distinct pathways occurring simultaneously, or alternatively, are implicated: clathrin-mediated endocytosis and clathrin independent phagocytosis^6^,^7^,^8^,^9^. Clathrin-mediated endocytosis is associated with internalization of small particles (<0.2 μm in diameter) and soluble aggregated molecules into cells via clathrin-coated pits that are formed by multiple accessory and adaptor proteins including dynamin, adaptor protein-2 (AP2) and epsin^10^. These contain ubiquitin-interacting motifs (UIM) that bind ubiquitin ligases, including Cbl, that cause receptor ubiquitination^10^,^11^,^12^. Heat shock cognate protein (HSC) 70 is a constitutive member of the heat shock protein family key in disassembly of the clathrin coat^13^,^14^. It facilitates fusion of the disassembled clathrin pit with early endosome where receptors are sorted for recycling, directly degraded by Cbl, or delivered to lysosomes^10^. Cbl-ubiquitinated proteins may also be recognized by hepatocyte growth factor-regulated tyrosine kinase substrate (HGS or HRS) and sorted from early endosomes to late endosomes for endo-lysosomal degradation^15^,^16^. Although not reported as part of the clathrin-mediated endocytosis cascade, tripartite motif-containing protein 21 (TRIM21), also known as E3 ubiquitin-protein ligase, was recently described as an intracellular Fc receptor that recognises cytoplasmic antibodies or immune complexes that escape from endosomes and promotes their proteasomal degradation^17^. Thus control of these processes by phosphorylation and dephosphorylation events may regulate the fate of endocytosed particles and their receptors. However, data describing the Tyr phosphorylation state of these molecules following FcγRI cross-linking is very limited. Moreover, mechanisms regulating dephosphorylation of these molecules, and their functional consequences, are unknown.

Excessive activation of FcγRI can induce unregulated inflammation leading to host tissue damage^18^, thus requires tight regulation. The leukocyte immunoglobulin-like receptor B4 (LILRB4), an ITIM-containing inhibitory receptor on the surface of mono-myeloid cells, is emerging as a key modulator of activation^19^,^20^. Co-ligation of LILRB4 with FcγRI on monocytes potently inhibits cytokine production through recruitment of Src homolog-containing phosphatases (SHP-1, SHP-1-like), resulting in Tyr dephosphorylation of a cascade of protein tyrosine kinases^19^. We proposed that LILR4B4 may regulate Fc-receptor-dependent endocytosis/phagocytosis by dephosphorylating key signalling molecules, akin to its inhibitory effects on FcγRI-mediated cytokine production^19^,^21^,^22^. Our aim was to identify Tyr phosphorylated proteins following FcγRI cross-linking on monocytes and determine whether their phosphorylation was modified by LILRB4 ligation.

Lysates of FcγRI-activated THP-1 cells were immunoprecipitated using anti-pTyr antibody and in-gel tryptic digested peptides sequenced by mass spectrometry (LC-MS/MS). Mascot Search output identified 25 high confidence Tyr-phosphorylated proteins and analysis using the Ingenuity Pathway analysis (IPA) revealed that the most significantly affected pathway was clathrin-mediated endocytosis, followed by the Fc receptor-mediated phagocytic pathway. Phosphorylation of 7 key candidate proteins, including the Fc receptor common γ-chain, Syk, clathrin, E3 ubiquitin protein ligase Cbl (a member of Cbl family), HGS, HSP70 and TRIM21 were confirmed by Western blotting and immunoprecipitation. Importantly, co-ligation of LILRB4 with FcγRI caused significant Tyr dephosphorylation of these proteins, except for HSP70, and significantly suppressed uptake of antibody-opsonised bacterial particles. Unexpectedly, co-ligation of FcγRI with LILRB4 further increased Tyr phosphorylation of HSP70. Taken together, the results suggest that Tyr phosphorylation may play a critical role in Fc receptor-dependent endocytosis/phagocytosis and that LILRB4 modulates these functions by dephosphorylating key molecules including the upstream Fc receptor common γ chain, Syk and clathrin, as well as downstream molecules including Cbl, HGS and TRIM21.

## Results

### Increased global Tyr phosphorylation in THP-1 lysates after surface cross-linking of FcγRI

**Figure 1:**
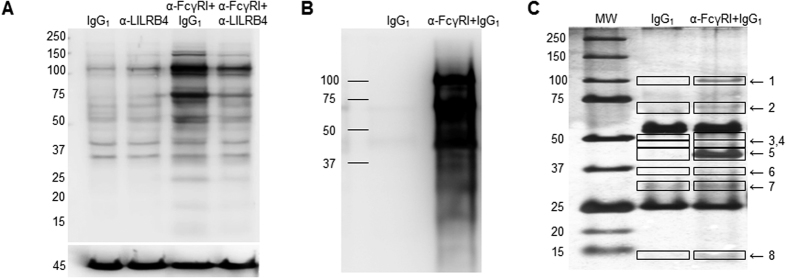
Cross-linking of FcγRI induced Tyr phosphorylation of multiple proteins that were reduced upon co-ligation with LILRB4. (**A**) Representative Western blot using anti-pTyr mAb showing marked increases in multiple Tyr phosphorylated proteins in total cell lysate from THP-1 cells ligated with specific mouse anti-FcγRI+IgG1 compared with cells treated with IgG1 alone. Tyr phosphorylated proteins in cells following co-ligation with anti-LILRB4 mAb and anti-FcγRI Ab were markedly reduced and more similar to those in cells treated with anti-LILRB4 mAb alone. The lower panel shows the same membrane stripped and re-probed with anti-β actin Ab confirming comparable protein loading (n = 3 experiments using different batches of THP-1 cells). Migration of the molecular weight markers is shown on the left. (**B**) Immunoprecipitation using anti-pTyr mAb (4G10) followed by Western blotting with biotinylated anti-pTyr mAb-100 mAb showed multiple strongly phosphorylated proteins in lysates from THP-1 cells cross-linked with specific mouse anti-FcγRI mAb but not control IgG_1_ (n = 3 separate experiments). (**C**) Silver staining of SDS-PAGE gels loaded with anti-pTyr mAb-precipitated lysates from FcγRI cross-linked cells indicated enrichment of 8 components that separated at approximately 100, 70, 50, 47, 43, 35, 30 and 14 kDa, compared to precipitates from control IgG_1_-treated cells. Bands at 53 and 25 kDa that are evident in each sample likely represented the heavy and light chains of antibodies used for immunoprecipitation and were not sequenced (representative of 3 independent experiments using different batches of THP-1 cells). Mascot Search output of peptides sequenced by LC-MS/MS of the 3 experiments combined identified 80 specific hits of 25 Tyr phosphorylated candidate proteins (Mowse score > 50, p 0.05; >3 peptide matches) (see Table 1).

Western blotting with a pan anti-pTyr MAb showed that cross-linking of surface FcγRI with specific mouse mAb followed by goat anti-mouse secondary antibody in suspension markedly increased Tyr phosphorylation of multiple proteins above those seen when cells were treated with IgG1 alone. The intensity of reactivity was markedly reduced in lysates from cells that had been co-ligated with anti-FcgR1 and anti-LILRB1 (Fig. 1A). Immunoprecipitation using anti-pTyr mAb showed marked enrichment of phosphorylated proteins in lysates of FcγRI-cross-linked cells compared with those treated with IgG1 alone (Fig. 1B). Silver staining of SDS-PAGE gels loaded with anti-pTyr mAb-precipitated lysates from IgG1 and anti-FcγRI+IgG1 cross-linked cells showed enrichment of 8 bands that separated at 100, 70, 50, 47, 43, 35, 30 and 14 kDa compared to components separated from IgG1-treated cell immunoprecipitates (Fig. 1C). The 8 bands were excised and peptides sequenced by Nano LC-MS/MS. The Mascot Search output of 3 combined experiments identified 80 hits that comprised 25 Tyr-phosphorylated candidate proteins in peptides sequenced from lysates of anti-FcγRI+IgG1 cross-linked cells that were not identified in IgG_1_ control-treated cells (Mowse score > 50, p = 0.05; >3 peptide matches) (Table 1).

### Ingenuity Pathway Analysis (IPA) of Tyr phosphorylated proteins generated by FcγRI cross-linking on THP-1 cells

**Figure 2:**
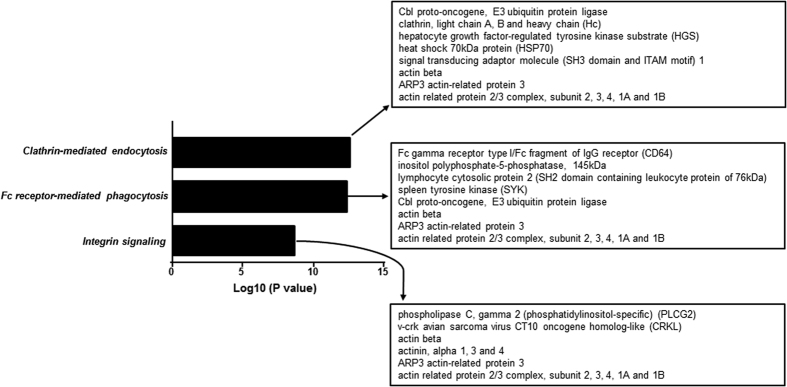
Pathway analysis of Tyr-phosphorylated proteins after FcγRI-cross-linking. Ingenuity Pathway Analysis of the 80 enriched peptides from the combined 3 experiments showed that clathrin-mediated endocytosis was predicted to be the most enriched signalling pathway (p =  2.19 × 10^−13^) followed by FcγRI-mediated phagocytosis and integrin signalling (p = 4.11 × 10^−13^ and p = 1.88 × 10^−9^) respectively.

To examine potential signalling pathways enriched by antibody cross-linking of FcγRI, the 80 hits identified by Mascot analysis were imported into to the IPA software. Clathrin-mediated endocytosis was predicted as the most enriched signalling pathway (p = 2.19 × 10^−13^) (Fig. 2). The second most enriched pathway was Fc receptor-mediated phagocytosis in macrophage and monocytes (p = 4.11  × 10^−13^), a known pathway activated after FcγRI cross-linking^1^,^5^ (Fig. 2). The key phosphorylated proteins identified in one or both pathways were Fc receptor γ chain, Syk, clathrin, Cbl, HGS, STAM1/2, HSP70, TRIM21, actin, actin-related proteins, actinin-4, tubulin and actin-binding proteins (Fig. 2 and Table 1). This is the first report to show simultaneous Tyr phosphorylation of these proteins following FcγRI cross-linking. Although not reported to date in monocytes in the context of FcγRI cross-linking, other Tyr-phosphorylated peptides identified matched proteins involved in cellular activation, migration and differentiation^23^,^24^,^25^,^26^ (Table 1). These included phospholipase C gamma 2, mitogen-activated protein kinase 9, hematopoietic cell-specific Lyn substrate 1, SH2 domain containing leukocyte protein of 76 kDa, linker for activation of T cell family member 2, Twinfilin-1, docking protein 2, protein tyrosine phosphatase 18, phosphatidylinositol 3, 4, 5-triphosphate 5-phosphatase 1 (SHIP), protein phosphatase 1 gamma, 1-phosphatidylinositol 4, 5-biphosphate phosphodiesterase gamma 2, Toll-like receptor 6, Crk-like protein, Coatomer subunit epsilon and ubiquitin-40S ribosomal protein s27a (Table 1).

### Regulation of FcγRI-mediated Tyr phosphorylation of Fc receptor γ chain, clathrin, Cbl, HGS, HSP70, TRIM21 and Syk by LILRB4

**Figure 3:**
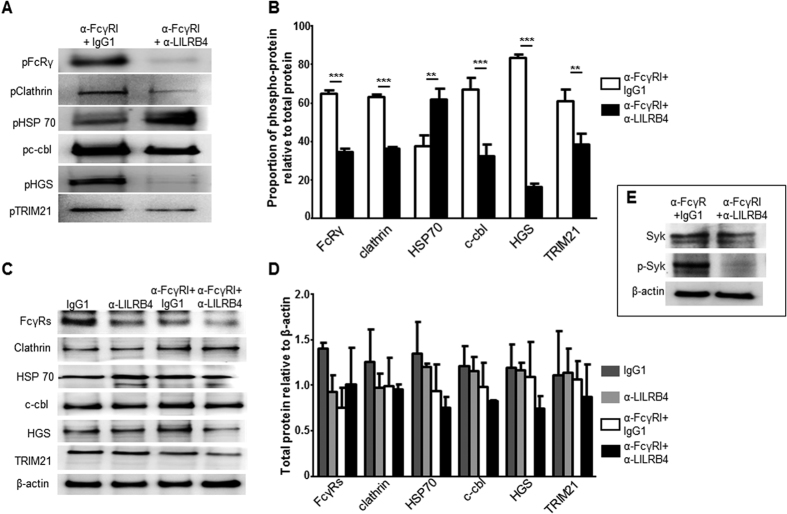
Co-ligation of LILRB4 with FcγRI suppressed Tyr phosphorylation of multiple proteins involved in clathrin-mediated endocytosis, and of TRIM21. (**A**) Representative immunoprecipitation of THP-1 cell lysates using anti-pTyr mAb (4G10) followed by Western blotting using selected antibodies showed abundant Tyr phosphorylation of FcγR s, clatherin, HSP70, Cbl, HGS and TRIM21 in cells co-ligated with anti-FcγRI+IgG_1_ control mAb validating LC-MS/MS data. Importantly, LILRB4 co-ligation with FcγRI markedly reduced Tyr phosphorylation of all proteins except for HSP70 (n = 3). (**B**) Summary of densitometry of bands from 3 independent experiments showed significant reduction of FcγRs, clatherin, Cbl, HGS and TRIM21 phosphorylation, but not HSP70, in THP-1 cells co-ligated with anti-FcγRI and anti-LILRB4 mAbs, compared to cells co-ligated with anti-FcγRI and negative control mAb (n = 3, **p < 0.01; ***p < 0.001). (**C**) Representative Western blotting of total cell lysates showed that co-ligation of FcγRI with LILRB4 did not alter the total amounts of any of the above proteins when compared to co-ligation of FcγRI+IgG_1_ control, ligation of LILRB4 alone or treatment with IgG_1_ control alone; the lower panel is the same membrane stripped and re-probed with anti-β actin Ab, confirming comparable protein loading. (**D**) Summary of densitometry analysis of 3 independent experiments showed no significant differences in total FcγRs, clatherin, HSP70, Cbl, HGS and TRIM21 in THP-1 cells within the 4 different treatment groups (n = 3). Full image of the Western blots is shown in Supplementary Fig. 1. (**E**) Western blotting of cell lysates from FcγRI cross-linked cells showing increased Tyr-phosphorylated Syk that was markedly reduced upon co-ligation with LILRB4, confirming our earlier finding^21^ and validating current LC-MS/MS data (Fig. 2) (n = 1).

Immunoprecipitation of cell lysates using anti-pTyr mAb followed by Western blotting using antibodies against the FcRγ chain, clatherin, Cbl, HGS, HSP70 and TRIM21 showed Tyr-phosphorylation of all these proteins in cells activated through FcγRI cross-linking (Fig. 3A) (supplementary Fig. 1), validating the LC-MS/MS data. Importantly, phosphorylation of all proteins except HSP70 was markedly suppressed upon co-ligation of FcγRI with LILRB4 (Fig. 3A). Semi-quantitative analysis indicated that LILRB4 significantly reduced Tyr phosphorylation of HGS by an average of 80.2%, Cbl by 51.3%, clathrin by 45.5% and TRIM21 by 36.9% (Fig. 3B; n = 3). In contrast, co-ligation of FcγRI with LILRB4 significantly enhanced HSP70 phosphorylation by 36.4%, suggesting selective suppressive effects by LILRB4. Similarly, Western blotting of cell lysates from FcγRI cross-linked cells caused Tyr-phosphorylation of Syk that was markedly reduced up on co-ligation with LILRB4 (Fig. 3E), further validating the LC-MS/MS data and confirming our previous finding^19^. As expected, the brief cross-linking/co-ligation protocols used in this study did not affect the total amounts of any of the above proteins (Fig. 3C,D) (Supplementary Fig. 1).

### LILRB4 suppressed uptake of antibody-opsonised bacterial particles

**Figure 4:**
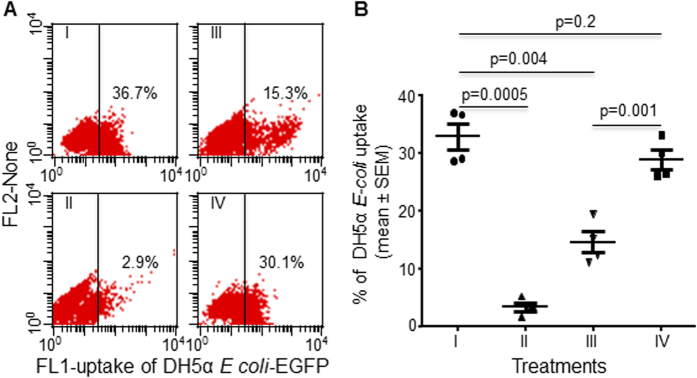
Ligation of LILRB4 significantly suppressed Fc receptor-dependent uptake of antibody-opsonised. *E. coli* by differentiated THP-1 cells. (**A**) Representative dot plot showing uptake of Ab opsonized EGFP-expressing DH5α *E-coli* particles by 36.7% of PMA-differentiated THP-1 cells (I) that was reduced by 90% when surface FcγRI was blocked by pre-incubating cells with 20 μg/ml anti-FcγRI mAb (II), but not by cells pre-incubated with negative control IgG_1_ mAb, indicating Fc-receptor dependent endocytosis/phagocytosis (n = 4). Ligation of LILRB4 with mouse anti-LILRB4 mAb followed by goat anti-mouse secondary Ab reduced uptake of Ab opsonized EGFP-expressing DH5α *E-coli* particles by >50% (III) when compared with non-ligated cells (I). Ligation of control mouse anti-MHC-I mAb (IV) or negative control mouse mAb had little effect on uptake, confirming specific LILRB4-mediated suppression. (**B**) Summary analysis of 4 independent experiments presenting mean percentages (±SEM) showing that numbers of PMA- differentiated THP-1 cells that took up Ab-opsonized EGFP-expressing DH5α *E-coli* particles were significantly less if cells were pre-incubated with anti-FcγRI mAb (p = 0.0005), and markedly less following Ab ligation of surface LILRB4 (p = 0.004), but not Ab ligation of MHC-I (p = 0.2).

For this experiment we used PMA-differentiated THP-1 cells because these have superior phagocytic activity. The mean percentage of these cells that took up antibody-opsonised fluorescent DH5α *E-coli* particles identified by flow cytometry, was 32.9 ± 2.3%. Uptake was significantly reduced to 3.5 ± 0.74% when surface FcγRI was blocked using a specific anti-FcγRI mAb (blocked by ~90%; p = 0.0005, n = 4; Fig. 4B), but not when cells were treated with an irrelevant negative control mAb (36.7 ± 4.6%) confirming involvement of FcγRI-dependent endocytosis/phagocytosis. Ligation of surface LILRB4 using an anti-LILRB4 mAb, followed by goat anti-mouse secondary antibody, significantly suppressed uptake of opsonised DH5α *E-coli* by up to 55% (p = 0.004, n = 4; Fig. 4B). In contrast, co-?ligation of surface MHC-I using anti-MHC-I mAb did not significantly alter uptake of opsonised DH5α *E-coli* particles when compared with non-ligated cells (28.9 ± 1.7% versus 32.9 ± 2.3% Fig. 4B), suggesting specific suppression of endocytosis/phagocytosis by LILRB4.

## Discussion

Reversible Tyr phosphorylation of proteins in eukaryotes is critical in regulating intracellular signalling pathways involved in cellular activation, growth, proliferation, differentiation migration and gene transcription^27^,^28^. Immune-complex mediated activation of FcγRI on innate immune cells is essential for protection against bacterial infection. Activation of Tyr phosphorylation of selected upstream protein tyrosine kinases and downstream mitogen activated protein kinases result in production of pro-inflammatory cytokines, generation of an oxidative burst, and/or triggering of endocytosis/phagocytosis^1^,^5^. In this study we immunoprecipitated total Tyr phosphorylated proteins from lysates of THP-1 that had been cross-linked with specific anti-FcγRI or control IgG mAb and found marked enrichment of phosphorylated proteins in cells activated with the specific mAb. Peptide sequencing by Nano LC-MS/MS identified 80 candidate peptides that were significantly modified, representing 25 Tyr-phosphorylated proteins. Pathway analysis predicted that Ty-phosphorylation of proteins mediating clathrin-mediated endocytosis and Fc receptor-mediated phagocytosis were the most affected. The most prominent phosphorylated proteins included Fc receptor γ chain, Syk, clathrin, Cbl, HGS, STAM1/2, HSP70, TRIM21, actin, actin-related proteins, actinin-4, tubulin and actin binding proteins. This is the first study to demonstrate the simultaneous Tyr phosphorylation of these proteins during FcγRI-mediated monocyte activation. This is particularly novel for clathrin, Cbl, HGS and HSP70, key molecules involved in the clathrin-mediated endocytosis^10^,^13^,^14^ and for TRIM21, a ligase recently identified as high affinity intracellular Fc receptor^17^,^29^ which is critical for ubiquitination and degradation of antibody-opsonised viruses^17^. We validated the data generated by LC-MS/MS using a combination of Western blotting and immunoprecipitation and confirmed that these molecules were indeed Tyr phosphorylated following cross-linking of FcγRI on the surface of THP-1 cells. Moreover, we confirmed our previous finding^19^ of Tyr phosphorylation of Syk in response to FcγRI cross-linking and identified Tyr phosphorylation of the intracellular tyrosine-based activating motifs (ITAMs) of the common γ chain of Fc-receptors that is upstream of Syk, directly linking for the first time, these two critical signalling events. Functionally, Tyr phosphorylation of Syk after FcγRI cross-linking on monocytes promotes cytokine production^19^,^21^,^22^ and Syk phosphorylation has been associated with increased phagocytosis of opsonised pathogens^30^ and polybeads,^31^ and enhanced endocytosis of immune complexes^32^. Here, we found that co-ligation of FcγRI with LILRB4 that contains intracellular tyrosine-based inhibitory motifs (ITIMs), significantly reduced phosphorylation of the ITAMs of the common γ chain, and of Syk. Suppression of Syk phosphorylation is consistent with our previous report showing that LILRB4, through recruitment of SHP-1-like phosphatase, dephosphorylated Syk and multiple downstream protein tyrosine kinases including Lck, LAT and Erk in THP-1 cells leading to suppressed cytokine production^19^. Importantly, we show here that LILRB4 ligation significantly inhibited FcγRI-dependent uptake of antibody-opsonised *E. coli* particles. One mechanism may involve dephosphorylation (inactivation) of the common γ chain ITAMs and Syk, similar to its inhibitory effects on FcγRI-mediated cytokine production^19^,^21^,^22^.

**Figure 5:**
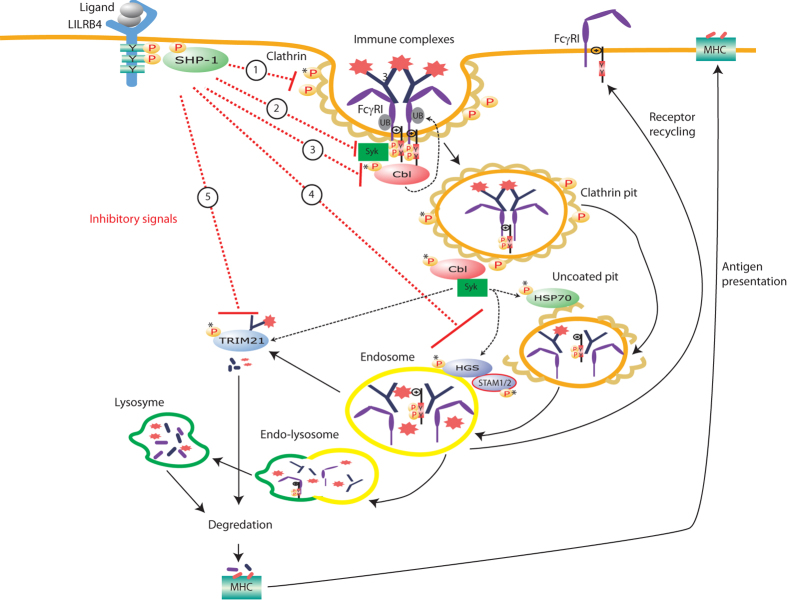
Schematic diagram suggesting possible roles of Tyr phosphorylation of key molecules involved in clathrin-mediated endocytosis of FcγRI and ligands, and their regulation by LILRB4. Cross-linking of FcγRI by immune-complexes causes Tyr phosphorylation of the ITAMs of its common γ chain and binding of pSyk transduces activating signals. This simultaneously initiates phosphorylation of clatherin that causes lateral diffusion of receptor-ligand complexes to clathrin-coated pits, membrane invagination and generation of clathrin-coated vesicles, and/or initiates phosphorylation of Cbl that may directly ubiquitinate the receptor. Phosphorylated Cbl triggers phosphorylation of HSP70 that facilitates un-coating of the vesicles, a precondition for vesicles to fuse with early endosomes and release ligands. The released receptors are transported to either the late endosome and/or lysosome for proteosomal and/or lysosomal degradation or are recycled to the cell surface. The immune complexes in the endosome are either directly degraded by Cbl, or delivered to the lysosome by phosphorylated HGS-STAM 1/2 complex for final degradation. During transfer, immune complexes that escape the endosome are recognised by phosphorylated TRIM21 for proteasomal degradation. Co-ligation of FcγRI with LILRB4 may recruit phosphatases such as SHP-1 to its ITIMs that subsequently dephosphorylate (deactivate) the key molecules including clathrin (1), FcγRI and Syk (2), Cbl (3), HGS and STAM 1/2 (4) and TRIM21(5). These effects may reduce cellular activation and/or suppress receptor/ligand endocytosis. *New Tyr phosphorylated and dephosphorylated proteins identified in this study.

Clathrin-mediated endocytosis is an important energy efficient pathway of pathogen/antigen clearance by innate immune cells as an alternate to phagocystosis^7^,^9^,^33^,^9^,^33^. It is also an important mechanism of endogenous surface ligand/receptor internalisation, ubiquitination and recycling/degradation^7^,^9^,^33^. Clathrin-mediated endocytosis may share some upstream signalling molecules with phagocytosis but unlike to phagocytosis, it is associated with internalisation of small particles (<0.2 μm in diameter) and soluble aggregated molecules^10^. During endocytosis, receptor-ligand or antibody-antigen complexes are first ubiquitinated and internalised into clathrin-coated pits assembled within AP2, dynamin, epsin and related molecules followed by HSP70-mediated un-coating of the pits and their endosomal sorting^10^,^13^,^14^. Receptors and ligands in the endosome are then either ubiquitinated and directly degraded by Cbl^10^,^11^,^12^,^17^ or the ubiquitinated molecules are delivered to lysosome by HGS/STAM1/2 complexes for lysosomal degradation^15^,^16^. Alternatively, the receptor in the endosomes is rapidly recycled while the ligands undergo endo-lysosomal degradation^10^. Although the involvement of clatherin, Cbl, HSP70, HGS and STAM in this pathway is generally accepted, whether Tyr phosphorylation/dephosphorylation regulate their functions, particularly during Fc-receptors mediated monocyte activation remain unexplored. There is limited evidence that Tyr phosphorylation of clathrin heavy chain promotes bacterial internalisation^34^ and that phosphorylation of Cbl is associated with receptor/ligand ubiquitination after receptor clustering in antigen presenting cells, T cells and B cells^35^,^36^. Tyr phosphorylation of HGS in Hela cells during epidermal growth factor (EGF)-mediated activation may be involved in intracellular receptor sorting and vesicle formation^37^. C-terminal Tyr phosphorylation of HSP70 is described as a switch that regulates co-chaperon binding in cancer cells and determines whether it facilitates protein folding, or directs proteins for ubiquitin-mediated degradation^38^. These functional observations collectively suggest that Tyr phosphorylation of these particular molecules may play critical roles in Fc receptor-dependent endocytosis of immune complexes. Here we show that co-ligation of LILRB4 with FcγRI significantly reduced FcγRI-mediated Tyr phosphorylation (activation) of clathrin, Cbl, HGS and STAM1/2 (Fig. 5). We propose that LILRB4 may inhibit Fc-receptor-dependent endocytosis of antigen-antibody complexes by promoting Tyr dephosphorylation (deactivation) of these key molecules. This proposal is consistent with its reported anti-inflammatory and immunosuppressive properties^19^,^39^,^40^,^41^ and our demonstration that it significantly suppressed FcγRI-dependent endocytosis/phagocytosis of antibody-opsonised *E. coli* particles (Fig. 4). In contrast to these dephosphoryating events, co-ligation of LILRB4 with FcγRI significantly enhanced FcγRI-mediated HSP70 Tyr phosphorylation by 36.4%, indicating selective effects. Whether enhanced HSP70 phosphorylation by LILRB4 regulates ubiquitin-mediated degradation of antibody-opsonised bacterial, particles and/or the sorting of the internalised Fc-receptors, requires further investigation.

TRIM21 is described as an important high affinity intracellular Fc receptor implicated in elimination of antibody-bound intracellular viruses^17^, although little is known about interactions that link extracellular antibody-bound pathogen to intracellular TRIM21. Here we found that surface cross-linking of FcγRI that uses a method that mimics antibody-antigen complexes, promoted strong phosphorylation of TRIM21. Thus FcγRI might be the missing link between activation by extracellular antibody-bound pathogens (antigen) and their intracellular Fc receptor (TRIM21) that may also function as a novel downstream pathway in Fc receptor-dependent endocytosis of immune complexes. Interestingly, TRIM21 is Tyr phosphorylated in TLR3 or TLR4-stimulated monocytes and macrophages and is suggested to activate downstream TLR-mediated signalling^42^. The significant dephosphorylation of TRIM21 caused by LILRB4 ligation shown by us, may therefore indicate functional deactivation of this molecule when FcγRI-induced monocyte activation is moderated by LILRB4.

In conclusion, results presented here suggest that Tyr phosphorylation of the upstream common γ-chain, Syk, and clathrin and the downstream molecules such as TRIM21 may be critical in Fc-receptor (FcγRI)-dependent endocytosis/phagocytosis of antibody-opsonised particles. Importantly, LILRB4 may regulate this important innate immune function by promoting mechanisms that dephosphorylate these proteins.

## Materials and Methods

### Cells and antibodies

Human monocytic leukemic THP-1 cells (ATCC clone TIB-202, Manassas, VA, USA) were cultured in RPMI 1640 supplemented with 2 mM L-glutamine, 10% heat-inactivated fetal bovine serum (FBS), 100 U/ml penicillin, 100 μg/ml streptomycin, 1 mM sodium pyruvate, 10 mM HEPES and 0.1% β-mercaptoethanol (all from Life Technologies) and 20 mM sodium bicarbonate (Sigma-Aldrich) at 37 °C with 5% CO_2_^19^. The following antibodies were used for flow cytometry and/or cross-linking/co-ligation experiments; anti-LILRB4 (kindly donated by Dr. Luis Borges, Amgen Inc), anti-FcγRI (R&D System, Minneapolis, MN, USA), IgG_1_ negative control (Sigma-Aldrich) mouse primary mAbs and F (ab’)_2_ fragment goat anti-mouse IgG (Fc-specific) secondary Ab (Jackson ImmunoResearch, West Grove, PA, USA). Mouse anti-pTyr mAb (clone 4G10; Upstate Biotechnology, Lake Placid, NY, USA) was used for immunoprecipitation. The following antibodies were used for Western blotting: biotinylated mouse α-pTyr-100 mAb (Cell Signaling, Danvers, MA, USA), mouse anti-human clathrin (Thermo Fisher Scientific, Waltham, MA, USA), mouse anti-HSP70 (Stressgen/Enzo Life Sciences, Farmingdale, NY, USA), rabbit anti-HGS (Thermo Fisher Scientific), rabbit anti-Cbl (Sigma-Aldrich), rabbit anti-FcγRs (Upstate Biotechnology Inc, Lake placid, NY, USA), rabbit anti-Syk (Cell Signaling), rabbit anti-pSyk (Tyr 525/526) (Cell Signaling), mouse anti-β-actin (Sigma-Aldrich), and mouse anti-human TRIM21 mAb (R&D System) in-house labelled with biotin using lightning-link^TM^ biotin conjugation kit (Innova Biosciences, Babraham, Cambridge, UK), primary antibodies, and HRP-conjugated goat anti-mouse or goat anti-rabbit secondary antibodies, and HRP-conjugated streptavidin (all from Bio-Rad, Gladesville, NSW, Australia). Mouse anti-MHC-I mAb (anti-HLA-ABC) (BD Biosciences, Mountain View, CA, USA) was used as relevant surface binding control Ab in detection of antibody opsonised bacteria uptake upon LILRB4 ligation.

### Immunoprecipitation of tyrosine-phosphorylated proteins after FcγRI cross-linking and identification of phosphorylated peptides by mass spectrometry

A total 2 × 10^7^ THP-1 cells in 50 μl cross-linking buffer (CLB; RPMI supplemented with 10 mM HEPES, 1 mM MgCl_2_, 0.1 mM CaCl_2_ and 0.1% bovine serum albumin CLB) were incubated with 5 μg/ml IgG_1_ control, anti-FcγRI, anti-LILRB4 or anti-FcγRI+anti-LILRB4 mAbs for 15 min at RT. Cells were washed in 1 ml CLB then resuspended in 100 μl CLB, then cross-linked with 15 μg/ml goat anti-mouse IgG (Fc-specific) secondary antibody for 90 sec at RT. Cell activation was stopped by adding cold PBS, cells harvested by centrifugation at 4 °C then lysed with cold Western lysis buffer containing 150 mM NaCl, 50 mM Tris-HCl (pH 8.0), 5 mM EDTA and 1% NP-40, freshly-made protease inhibitors (2 mg/ml; Roche Applied Science) and 10 μM sodium pervanadate (Sigma-Aldrich). After vortexing for 1 min, samples were incubated on ice for 30 min then supernatants collected by centrifugation at 20,000xg for 10 min at 4 °C. Tyr-phosphorylated proteins were immunoprecipitated using 5 μg/ml anti-pTyr mAb (clone 4G10) at overnight 4 °C then incubated with goat anti-mouse secondary antibody conjugated to Sepharose beads (10 μg/ml; Zymed Laboratories Inc., San Francisco, CA, USA) for 2 hrs at 4 °C. Bead-bound proteins were washed once with 1 ml cold dilution buffer (0.1% Triton X-100 in TSA buffer pH 8.0; 0.01 M Tris buffer, 0.14 M NaCl, 0.025% NaNa_3_), two washes with TSA and a single wash with 50 mM Tris buffer pH 6.9. Beads were then resuspended in Tricine gel loading buffer containing 10 mM dithiothreitol, heated for 5 min at 100 °C and supernatants resolved in 10% Tris-Tricine SDS-PAGE gels under reducing conditions then silver-stained. Specific silver-stained bands were excised and Tyr-phosphorylated proteins were identified by Nano Liquid Chromatography tandem Mass Spectrometry (Nano LC-MS/MS) as described^43^,^44^. Bands excised from lanes loaded with immunoprecipitates of irrelevant IgG_1_-cross-linked THP-1 cells were used as negative controls. Peak lists of MS/MS data were generated using Mascot Daemon/extract_msn (Matrix Science, London, England, Thermo) were interrogated using Mascot version 2.1 (http://www.matrixscience.com) and searched against *Homo sapiens* proteins in the Swissprot protein database (version 80). Precursor tolerances were 4.0 ppm and product ion tolerances were ± 0.4 Da and acceptable cut-off scores for individual MS/MS spectra were set to 20. Specific phosphorylated peptides identified in FcγRI cross-linked cells, but not in cells treated with control IgG_1_ from 3 independent experiments, were combined and uploaded onto Ingenuity Pathway Analysis software version 24718999, used to predict the most significantly-enriched pathways (IPA^®^; www.qiagen.com/ingenuity, QIAGEN, Redwood City, CA). Alternatively, proteins were transferred onto PVDF membranes (0.2 μm pore size; Millipore, Bayswater, VIC, Australia) for Western blotting using 1 μg/ml biotinylated α-pTyr-100 mAb.

### Validation of enriched Tyr phosphorylated proteins by immunoprecipitation and Western blotting and regulation by LILRB4

Six proteins including the common γ chain of the Fc receptor, clathrin, Cbl, HGS, TRIM21 and HSP70 had multiple Tyr phosphorylated peptides with high Mascot scores following FcγRI cross-linking. Four of these are reported to be involved in clathrin-mediated receptor endocytosis, although this was the first demonstration of their simultaneous phosphorylation. Hence, results were validated, and their regulation by LILRB4 examined using a combination of immunoprecipitation and Western blotting. In brief, 2 × 10^7^ THP-1 cells were activated via FcγRI cross-linking with or without LILRB4 co-ligation [21], lysates immunoprecipitated using anti-pTyr mAb (4G10) followed by serial Western blots using antibodies against FcR common γ chain, clathrin, Cbl, HGS, TRIM21 or HSP70. In separate experiments, Western blotting using anti-pTyr (4G10) was performed using 20 μg total lysates to detect global protein phosphorylation; membranes were re-probed with 1 μg/ml mouse anti-β-actin mAb to confirm equal protein loading. For detection of Syk and p-Syk proteins, 20 μg of total lysates were serially Western blotted using rabbit anti-pSyk Ab followed by rabbit anti-Syk Ab and mouse anti-β-actin mAb.

### Detection of uptake of antibody opsonised bacteria particles by differentiated THP-1 cells and modulation by LILRB4

To determine uptake of antibody-opsonised bacteria by phorbol 12-myristate 13-acetate (PMA) differentiated THP-1 cells a modified Fc-receptor dependent endocytosis/phagocytosis assay was developed. In brief, desired numbers of THP-1 cells were cultured in RPMI complete medium containing 100 ng/ml PMA (Sigma-Aldrich) at 37 °C in 5% CO_2_ for 3 days. Differentiation was confirmed by assessing morphological changes; larger, partially adherent cells, vesicular with ruffled edges and non-replicating. Enhanced green fluorescent protein (EGFP) expressing DH5α *E. coli* (Addgene, Cambridge, MA, USA) (10^7^ cells/ml PBS) were killed by freeze-thawing twice and pellets incubated with 5 μg/ml goat anti-DH5α *E. coli* in PBS (Abcam 25823, Melbourne, VIC, Australia) for 2 hrs at 37 °C. The opsonised particles were added to 2 × 10^5^ PMA- differentiated THP-1 cells in 400 μl CLB, at an estimated bacteria to cell ratio of 10:1, and incubated at 37 °C for 4 hrs. Cells were then washed twice in 1.5 ml cold PBS containing 0.05% NaN_3_ and 1% bovine serum albumin, and re-suspended in 0.5 ml of 1% paraformaldehyde in PBS. The percentage of cells that took up bacterial particles was determined by flow cytometry. To confirm Fc-dependent uptake, FcγRI (the primary Fc receptor expressed on THP-1cells^19^) function was blocked by pre-incubating cells with 20 μg/ml anti-FcγRI mAb for 15 min at RT followed by a single CLB wash prior to addition of opsonised bacteria particles; 20 μg/ml irrelevant mouse negative control IgG_1_ mAb (Sigma-Aldrich) was used as a control. A total of 2 × 10^4^ events were acquired using BD FACSCalibur^TM^, and data analysed using Cell Quest software (BD Biosciences, Mountain View, CA, USA).

To assess the effect of LILRB4 ligation on the uptake of the opsonised DH5α *E. coli* particles, 2 × 10^5^ differentiated THP-1 cells were resuspended in 100 μl CLB and incubated with 10 μg/ml anti-LILRB4 mAb for 20 min at RT, followed by ligation using 15 μg/ml goat anti-mouse IgG_1_ (Fc-specific) secondary Ab at RT for 10 min, and a single CLB wash prior to addition of the *E. coli* particles. Mouse anti-MHC-I mAb (anti-HLA-ABC) was used as a relevant surface-binding control Ab.

### Statistical analysis

Fisher’s exact test was used to determine the most significantly enriched pathways as predicted by Ingenuity Pathway Analysis software. Western blots were semi-quantified by densitometry using ImageJ software (http://rsbweb.nih.gov/ij) and compared using one-way ANOVA with Dunnett post-test for multiple comparisons. The mean percentages of differentiated THP-1 cells that took up bacterial particles without LILRB4 ligation were compared with cells pre-ligated with anti-LILRB4, or treated with control anti-MHC-I mAb using two-tailed unpaired t-test. P values < 0.05 were considered statistically significant.

## Additional Information

**How to cite this article**: Park, M. *et al*. Leukocyte immunoglobulin-like receptor B4 regulates key signalling molecules involved in FcγRI-mediated clathrin-dependent endocytosis and phagocytosis. *Sci. Rep.* **6**, 35085; doi: 10.1038/srep35085 (2016).

## Supplementary Material

Supplementary Information

## Figures and Tables

**Table 1:** Mascot search results of mass spectrometric peptides sequencing of tyrosine phosphorylated proteins following FcγRI cross linking of THP-1 cells (n = 3).

*Band*	*Phosphoproteins detected in anti-FcγRI cross-linked but not control IgG1 treated THP-1 cells*	*Score*	*Peptide matched*	*Mass*
1	alpha actinin 1, sarcomeric (F-actin cross-linking protein)	5321	249	102
	alpha actinin 4	1927	77	102
	E3 ubiquitin-protein ligase CBL	964	71	99
	hepatocyte growth factor-regulated tyrosine kinase substrate (HGS)	231	17	86
	Toll-like receptor 6	116	12	92
	microtuble-associated protein 2	123	8	199
	ALG-2 interacting protein 1 (hp95/ program cell death 6 interacting protein)	112	3	96
	phosphatidylinositol 3,4,5-trisphosphate 5-phosphatase 1 (SHIP1)	98	3	133
2	protein Tyr kinase Syk	951	67	72
	heat shock cognate 71 kDa protein	831	46	70
	hepatocyte growth factor-regulated tyrosine kinase substrate (HGS)	209	31	86
	actinin, alpha 1	654	22	103
	protein SPY75 (hematopoietic cell-specific Lyn substrate 1)	289	11	54
	lymphocyte cytosolic protein 2 (SH2 domain containing leukocyte protein of 76 kDa)	287	11	60
	E3 ubiquitin-protein ligase CBL (proto-oncogene c-CBL, RING finger protein 55)	146	6	100
	Fc gamma receptor type I/ Fc fragment of IgG receptor, CD64)	76	4	42
	ATP-dependent DNA helicase II, 70 kDa subunit (G22P1)	64	3	70
3	elongation factor 1 alpha 1	402	30	50
	lymphocyte cytosolic protein 2 (SLP76)	293	27	60
	tripartite motif containing 21(TRIM21)	277	21	54
	Hematopoietic lineage cell-specific protein	261	13	53
	beta-actin	254	10	42
	tubulin beta chain	235	10	50
	signal transducing adaptor molecule 2B (STAM2)	232	8	58
	coronin, actin binding protein, 1C variant	230	7	53
	alpha actinin (4)	227	7	103
	docking protein 2	210	6	45
	protein Tyr kinase or Syk	175	7	72
	ARP3 actin-related protein 3 homolog	154	7	47
	signal transducing adaptor molecule 1 (STAM1)	83	4	59
	heat shock protein 70 kDa	93	3	70
	E3 ubiquitin-protein ligase CBL (Ring finger protein 55)	97	5	99
	unnamed human protein (IgG receptor Fc region II precursor)	78	3	35
	alpha-tubulin	74	3	50
4	ARP3 actin-related protein 3	1338	61	47
	mitogen-activated protein kinase 9	103	4	48
	actin non-muscle 6.2	504	19	41
	alpha actinin 4	95	3	102
	actin 7	91	3	37
	docking protein 2	79	3	45
	signal transducing adaptor molecule 2B (STAM2)	75	3	58
5	POTE ankyrin domain family member E	835	38	35
	actin related protein 2/3 complex subunit 1B (p41-ARC)	577	35	40
	F-actin capping protein alpha-1 subunit	480	29	32
	F-actin capping protein beta subunit (actin filament muscle Z-line)	208	4	30
	clathrin light chain (LCB3)	170	8	23
	histone cluster1, H1	83	8	23
	capping protein alpha (actin filament muscle Z-line, alpha 2)	141	6	33
	actin related protein 2/3 complex subunit 2	126	5	34
	EF-hand domain family, member D2	100	5	27
	protein tyrosine kinase (PTK9 or Twinfilin-1)	99	4	40
	protein phosphatase 1 gamma	97	3	37
	protein Tyr kinase (Syk)	80	4	72
	E3 ubiquitin-protein ligase CBL	70	3	100
6	actin related protein 2/3 complex subunit 2	515	38	34
	actin related protein 2/3 complex subunit 1B (p41-ARC)	434	17	41
	F-actin capping protein beta subunit (actin filament muscle Z-line, beta)	307	15	31
	EF-hand domain family, member D2	279	10	27
	linker for activation of T cells family member 2	133	6	31
	F-actin capping protein alpha-1 subunit	122	5	33
	high affinity immunoglobulin gamma Fc receptor I	97	5	32
	actin related protein 2/3 complex subunit 4 isoform a	96	4	20
	Crk-like protein	58	3	33
	coatomer subunit epsilon	56	3	34
7	spectrin beta chain, non-erythrocyte 4	221	14	28
	EF hand domain containing protein D2	311	17	26
	clathrin light chain B	106	13	25
	clathrin light chain A	91	8	27
	protein tyrosine phosphatase, non-receptor type 18	81	3	50
8	alpha actinin 4	2158	101	102
	actinin, alpha 1	1607	78	103
	clathrin heavy chain 1 (or KIAA0034)	375	25	191
	E3 ubiquitin-protein ligase CBL	354	13	100
	splicing factor proline/glutamine rich	288	16	76
	Ubiquitin-40S ribosomal protein s27a	219	17	17
	hepatocyte growth factor-regulated tyrosine kinase substrate	186	6	62
	high affinity immunoglobulin epsilon receptor subunit gamma	179	5	10
	protein Tyr kinase (p72 Syk)	95	4	96
	1-phosphatidylinositol 4,5-bisphosphate phosphodiesterase gamma-2 (PLCG2)	82	3	14
