# Supplementary figures and images for: Leukocyte immunoglobulin-like receptor B4 regulates key signalling molecules involved in FcγRI-mediated clathrin-dependent endocytosis and phagocytosis
Source: Sci Rep. 2016 Oct 11;6:35085. doi: 10.1038/srep35085 (PMC5057125; doi:10.1038/srep35085)

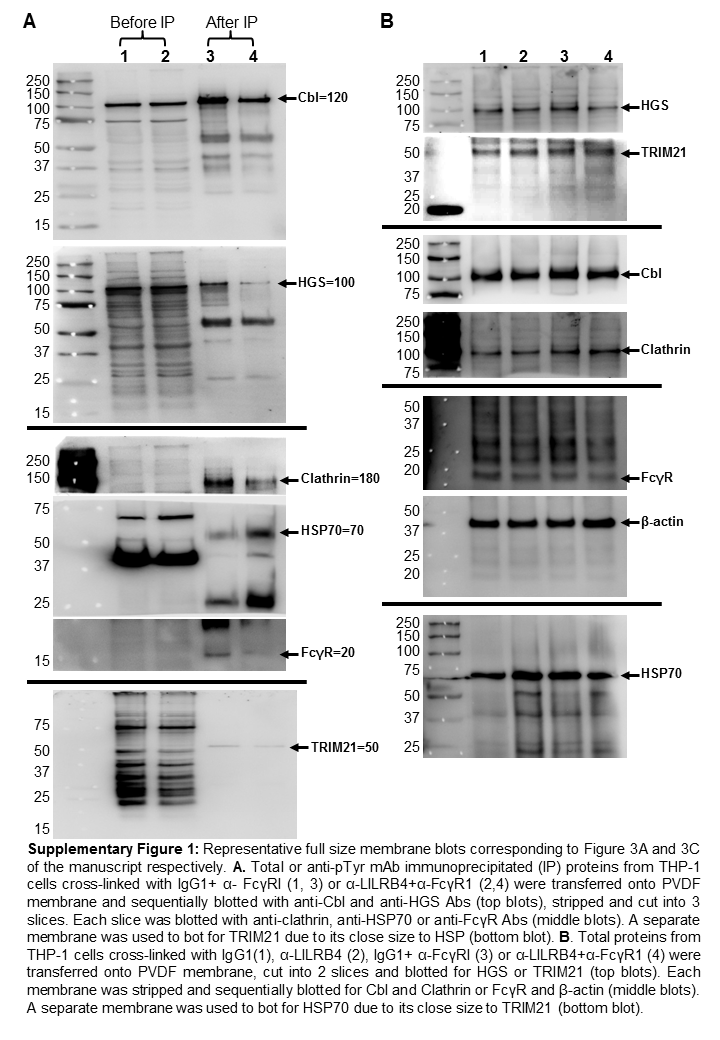

Supplement: Supplementary Information [file srep35085-s1.tiff]
